# The Role of Black Soldier Fly Larvae in Optimizing Performance and Amino Acid Composition in Thai Native Chicken

**DOI:** 10.3390/ani15091330

**Published:** 2025-05-05

**Authors:** Theeraphat Srikha, Padsakorn Pootthachaya, Warin Puangsap, Suphakon Pramotchit, Wuttigrai Boonkum, Chanon Suntara, Yupa Hanboonsong, Anusorn Cherdthong, Bundit Tengjaroensakul, Sawitree Wongtangtintharn

**Affiliations:** 1 Department of Animal Science, Faculty of Agriculture, Khon Kaen University, Khon Kaen 40002, Thailand; theeraphat.sr@kkumail.com (T.S.); padsakornp@kkumail.com (P.P.); suphakorn.p@kkumail.com (S.P.); wuttbo@kku.ac.th (W.B.); chansun@kku.ac.th (C.S.); anusornc@kku.ac.th (A.C.); 2 Department of Veterinary Public Health, Faculty of Veterinary Medicine, Khon Kaen University, Khon Kaen 40002, Thailand; warin.paun@kkumail.com (W.P.); btengjar@kku.ac.th (B.T.); 3 Network Center for Animal Breeding and Omics Research, Khon Kaen University, Khon Kaen 40002, Thailand; 4 Department of Entomology and Plant Pathology, Faculty of Agriculture, Khon Kaen University, Khon Kaen 40002, Thailand; yupa_han@kku.ac.th

**Keywords:** black soldier fly larvae, alternative proteins, production, Thai native chicken, Pradu Hang Dam Mor Kor 55

## Abstract

The objective of this study was to investigate the effects of black soldier fly larvae (BSFL) inclusion on the performance, economic return, carcass traits, meat quality, amino acid profile, and purine content in the breast meat of Thai native chickens (TNCs) (Pradu Hang Dam Mor Kor 55: PD) from 10 to 70 days of age. A total of **216 ten-day-old PD chickens (108 males and 108 females)** were randomly assigned to three dietary treatments with four replicates per treatment (18 birds per replicate, with a 1:1 male-to-female ratio). The treatments included (T1) control diet with soybean meal as the main protein source, (T2) 10% BSFL meal replacing soybean meal, and (T3) 12% BSFL meal replacing soybean meal. The results showed that BSFL inclusion had no significant effect on overall growth performance (*p* > 0.05), but it reduced abdominal fat and feed cost per gain while improving economic returns (*p* < 0.05). Additionally, BSFL increased some breast meat amino acid profiles, such as serine, glutamic acid, threonine, arginine, and lysine (*p* < 0.05), and increased total purine content (*p* < 0.05), although the values were still within the safe limit for human consumption. These results suggest that BSFL can be used as a sustainable alternative protein source in Thai native chicken diets without compromising product performance or quality.

## Simple Summary

This study evaluated the suitability of using black soldier fly larvae as a protein source in the diets of Thai native chickens. Black soldier fly larvae offer an environmentally friendly and nutritious alternative to conventional protein sources, providing benefits such as lower feed costs and increased economic returns. Although the use of black soldier fly larvae did not significantly affect growth performance, meat quality, or carcass characteristics, it showed potential to replace soybean meal and improve the amino acid profile of Thai native chickens. Additional studies are necessary to investigate ways to further enhance the economic value and sustainability of Thai native chicken production.

## 1. Introduction

Over the past several years, consumers worldwide have increasingly turned their attention to local and functional foods, expecting to obtain health benefits associated with specific compounds in these traditional foods [1,2]. Pradu Hang Dam Mor Kor 55 (PD), a selected strain of Thai native chicken (TNC) developed by Khon Kaen University, is known for its adaptability, disease resistance, and improved growth rate. In addition to these production traits, this strain has been reported to contain higher levels of health-promoting bioactive compounds such as L-carnitine, creatine, carnosine, and anserine, which may support antioxidant activity and physical performance in humans [3,4]. The reliance on imported soybeans for animal feed presents a significant challenge in managing production costs in Thailand’s poultry industry as a whole, including both commercial and native breeds such as PD. AS about 99% of the soybean demand is met through imports, fluctuations in global market conditions and prices can lead to increased and unpredictable feed costs [5]. This situation emphasizes the need to explore alternative protein sources for animal feed or develop more resilient strategies to mitigate the impact of price volatility on production cycles. Potential strategies include investing in the local production of alternative protein sources, improving feed efficiency, and researching more sustainable feed formulations that reduce dependence on imported soybeans [6]. Therefore, exploring new sources of protein is an important issue to consider in order to mitigate the risks associated with global instability.

Black soldier fly larvae (BSFL) are being increasingly explored as a new protein source for animal feed. They offer several advantages due to their rich nutritional profile, which is comparable to those of traditional protein sources such as soybean meal and fish meal [7]. The BSF is gaining attention due to its low production cost and environmental friendliness associated with the efficient conversion of organic waste into a usable source of protein and fat for animal feed [8,9,10,11,12]. Moreover, the black soldier fly can reproduce in tropical and warm countries and is found throughout Thailand.

Additionally, several studies have reported that the inclusion of BSFL in poultry diets can support growth performance by providing highly digestible proteins, essential amino acids, and medium-chain fatty acids, which improve feed efficiency, nutrient absorption, and fat metabolism, ultimately enhancing overall bird growth and health [13,14,15]. Previous studies have reported that incorporating BSFL into the diet of meat quails does not negatively affect the texture and flavor of breast meat. Moreover, it improves the contents of amino acids, including aspartic acid, glutamic acid, alanine, serine, tyrosine, and threonine, and raises the levels of both saturated and unsaturated fatty acids [16]. A similar study in broiler chickens using BSFL without oil found that this method helped to mitigate negative impacts on the fatty acid profile [8,9,17] and indicated that lauric acid, which is a major component of BSFL and differs from soybean sources, can help to reduce abdominal fat in broiler chickens. BSFL also has bioactive properties that inhibit pathogenic bacteria [18,19]. The potential of BSFL as poultry feed is evident in the literature [14]. However, the utilization of black soldier fly larvae in TNC, particularly the PD strain, has not been sufficiently studied. Important traits such as growth performance, carcass traits, meat quality, amino acid composition in breast meat, and economic return require further investigation. Confirming these aspects would provide strong evidence to support the use of black soldier fly larvae as a functional feed ingredient to enhance productivity and profitability in TNC production.

## 2. Materials and Methods

### 2.1. Animal Ethics

The Institutional Animal Care and Use Committee of Khon Kaen University has reviewed and approved the procedures and methods for this study (record no. IACUC-KKU-27/67) in accordance with the ethical guidelines for animal experimentation established by the National Research Council of Thailand.

### 2.2. Samle Preparation

The BSFL 100 kg were raised, cleaned, and euthanized at the Model House for Research and Production of Industrial Insects, Industrial Insect Division, Faculty of Agriculture, Khon Kaen University. The BSFL, following the method of Pornsuwan, et al. [20], were subsequently dried in a parabola dome on aluminum trays and evenly spread for 24 h until their dry matter (DM) content exceeded 90%. Oil extraction was performed using physical processes, and the larvae were finely ground with an MF 10 basic microfine grinder drive (IKA-Werke GmbH & CO. KG, Staufen, Germany) to pass through a 0.1 mm sieve to be used as an ingredient for TNC.

### 2.3. Animals and Experimental Design

This experiment will be conducted from June 2024–August 2024, 1-day-old PD, 216 (male to female ratio 1:1) by the Network Center for Animal Breeding and Omics Research, Faculty of Agriculture, Khon Kaen University, Thailand [3]. All chickens were raised and acclimatized with a controlled diet for 9 days before being randomly assigned to one of three experimental treatments. Each treatment consisted of 4 replicates, with each replicate comprising 18 chickens (9 males and 9 females). Three dietary treatments were used: the first, a control diet using soybean meal as the main protein source group (T1), and the second and third, where 10% (T2) and 12% (T3) of the soybean meal in the basal diet were replaced with BSFL, respectively. Rice hull was used as the bedding material. All birds were raised in pens with a total area of 4.20 m^2^. The chickens were exposed to 18 h of light and 6 h of darkness daily. The chickens were provided with pelleted feed and had unrestricted access to both the feed and water for the entire duration of the experiment. The composition of the feed and its chemical analysis are shown in Table 1. Each feed formulation meets the nutritional requirements of TNC up to the age of 70 days. The experimental period was divided into three phases: the starter phase (10–28 days), the grower phase (29–42 days), and the finisher phase (43–70 days). Chickens were provided with feed and water ad libitum throughout the rearing period.

### 2.4. Data Collection

#### 2.4.1. Growth Performance

The performance was monitored throughout the research. Individual body weights were recorded at 10, 28, 42, and 70 days to determine the body weight gain (BWG). Feed intake was also recorded to calculate the feed conversion ratio (FCR). Additionally, mortality was recorded to determine the survival rate (SR). Subsequently, the production index (PI) was calculated using Equation (1):

PI = (BWG (kg/b) × SR × 100)/(Number of days of experiment × FCR)(1)

#### 2.4.2. Economic Aspects

Additionally, some of the data were used to calculate the economic returns, including feed cost per gain (FCG; Equation (2)), salable bird return (SBR; Equation (3)), net profit per bird (NPR; Equation (4)), and return on investment (ROI; Equation (5)) which were calculated using Equations (2), (3), (4), and (5), respectively.

FCG = FCR ×Feed price × BW(g)(2)

SBR = 2.48 (US) ×BW (kg)(3)

NPR = SBR − FCG(4)

ROI = NPR/FCG × 100(5)

#### 2.4.3. Carcass Quality

On the 70th day of the experiment, 48 chickens (4 birds per experimental unit) were fasted for 12 h. Individual body weights were recorded before euthanasia, and hot carcass weights were noted. Then, the chickens were dissected into various parts, including the wing, thigh, inner fillet, outer fillet, and drumstick. The weights of these parts were recorded to calculate carcass yield within 10 min.

#### 2.4.4. Meat Quality

The outer fillet was divided into two pieces (left and right). The left section was kept at 4 °C for analysis of meat quality. The color of the meat was measured using a portable colorimeter (CR-410 Series, Konica Minolta Sensing Inc., Tokyo, Japan). The CIE L*, a*, and b* color parameters were determined by averaging three readings taken from three different points on the breast meat surface. In this study, texture profile analysis (TPA) of the left breast meat was evaluated, following the methods outlined in previous studies [4]. Meat samples were cut into 1 cm × 1 cm × 1 cm cubes and double-compressed for analysis using a texture analyzer (model TA.HDplusC, Stable Micro Systems, Godalming, UK) equipped with a 50 mm cylindrical aluminum probe for TPA measurements. All texture-related parameters were automatically calculated and reported by Exponent software version 6.2.1.0 (Stable Micro Systems, Godalming, UK) for both types of analysis.

#### 2.4.5. Amino Acid Profile of Breast Meat

The right breast meat was sent under controlled conditions at −20 °C. The first portion was sent to the Scientific Instrument Center at Ubon Ratchathani University, Thailand. The amino acid profile was examined using an amino acid analyzer. (Hitachi L8900, Hitachi High Technologies Corporation, Tokyo, Japan).

#### 2.4.6. Purine Contents of Breast Meat

The second portion was sent to the breeding and omics laboratory, Northeastern Science Park Building, Khon Kaen University, Thailand. To find the amount of the contents of purine (adenine, guanine, hypoxanthine, and xanthine) in breast meat were determined using high-performance liquid chromatography (HPLC) (Shimadzu model LC20A, Tokyo, Japan) with an Asahipak GS-HQ 320HQ column, 300 mm × 7.5 mm, 6 μm, at a temperature of 35 °C. HPLC was performed using a mobile phase of 150 mM sodium phosphate buffer (pH 2.5) at a flow rate of 0.6 mL min^−1^, and the running time was 35 min, according to the method of Tantiyasawasdikul et al. [21]

### 2.5. Statistical Analysis

The data analysis was conducted utilizing one-way ANOVA through the general linear model (GLM) procedure in SAS [22] program, based on a completely randomized design to evaluate all parameters. The data were first tested for normal distribution using the Shapiro–Wilk test and assessed for homogeneity of variance with Levene’s test; outliers identified through these checks were excluded before analysis. Significant differences between treatment means were determined using Duncan’s multiple-range test, with statistical significance at *p* < 0.05. Where appropriate, orthogonal polynomial contrasts were applied to examine linear and quadratic trends associated with the increasing levels of dietary BSFL. The statistical model is as follows:

Y_ijk_ = µ + T_i_ + Σ_ij_ (6)

where Y_ijk_ = observation, µ = overall mean, T_i_ = BSFL levels (i = 0, 10, and 12%), and Σ_i j_ = the experimental error.

## 3. Results

### 3.1. Growth Performance

Table 2 illustrates the growth performance of indigenous Thai chickens. The inclusion of BSFL at levels of 10% and 12% in the diet, from days 10 to 28, resulted in an increase in FI. This relationship was quadratic (*p* < 0.0001), indicating that FI did not increase in direct proportion to the BSFL levels but, rather, increased with the inclusion of BSFL in the diet. This trend had a comparable impact on the FCR and PI (*p* = 0.0002 and 0.002, respectively). During the period from days 29 to 42, the chickens may have adapted to the experimental diet, resulting in no significant impact on performance (*p* > 0.05). Interest was renewed during days 43 to 70, during which a linear relationship was observed between the BSFL levels in the diet and FI (*p* < 0.05). During this period, FI increased with the levels of BSFL, although no effects were observed on other performance indices. The inclusion of BSFL at 10% and 12% levels in the diet did not adversely affect the BWG, FI, FCR, SR, and PI (*p* > 0.05) when considering the entire period from days 10 to 70.

### 3.2. Economic Aspects

The impact of BSFL on economic aspects is presented in Table 3. In calculating feed costs, BSFL prices were based on the industrial insect category provided by the Faculty of Agriculture at Khon Kaen University, with a price set at USD 0.43 per kilogram. This study found that the inclusion of BSFL significantly reduced the average feed price and FCG (*p* < 0.05). Higher proportions of BSFL in the diet also led to reduced production costs, confirmed via a polynomial analysis of the linear trend (*p* = 0.003), and impacted the NPR, which showed an increasing trend at a 12% inclusion level (*p* = 0.08). Moreover, higher BSFL levels positively influenced the ROI, as confirmed by the polynomial analysis of the linear trend (*p* = 0.005), with the highest returns observed at a 12% inclusion level (*p* < 0.05).

### 3.3. Carcass Quality

The impact of BSFL on carcass quality is detailed in Table 4. This study found that varying proportions of BSFL did not significantly affect overall carcass quality (*p* > 0.05), with the exception of abdominal fat. The inclusion of BSFL was found to significantly reduce abdominal fat (*p* < 0.05), particularly at a 12% inclusion level. This reduction in abdominal fat with higher BSFL levels was confirmed via a polynomial analysis of linear trend (*p* = 0.02).

### 3.4. Quality of Meat

Table 5 shows the effects of BSFL in the diet on the meat quality of TNC. The inclusion of BSF did not significantly impact the breast meat color (*p* > 0.05). Similarly, the texture profile analysis, a distinguishing feature of TNC, was not significantly affected by BSFL (*p* > 0.05).

### 3.5. Amino Acid Profile of Breast Meat

Table 6 presents the effects of the dietary inclusion of BSFL on amino acid content in breast meat. It was found that native chickens fed diets containing BSFL at 10% and 12% levels showed a significant increase in the accumulation of certain amino acids, including serine, glutamic acid, arginine, threonine, and lysine, and this relationship was quadratic (*p* < 0.05). However, the total amino acid content was not significantly affected (*p* > 0.05).

### 3.6. Purine Content of Breast Meat

The inclusion of BSFL in the diet significantly affected the purine content in the meat of Thai native chickens (Table 7) compared to the control diet. All experimental diets containing BSFL (10% and 12%) showed a marked increase in purine concentrations. Chickens fed diets with 10% and 12% BSFL had significantly higher total purine levels (*p* < 0.05), and this relationship was quadratic (*p* < 0.05). When changes were calculated as a percentage compared to the control group, the adenine content exhibited a slight increase in all treatment groups, with improvements of 16.25% (BSFL10%) and 16.92% (BSFL12%). The guanine content showed a similar moderate increase of 62.26% (BSFL10%) and 42.99% (BSFL12%) compared to the control diet. Notably, hypoxanthine exhibited the most substantial increase among all evaluated purine bases, rising by 361.61% (BSFL10%) and 671.42% (BSFL12%) relative to the control. Overall, the inclusion of BSFL in chicken diets consistently increased purine content, especially hypoxanthine, across all experimental groups.

## 4. Discussion

The impact on performance during the starter phase showed that BWG was not significantly different. However, the use of BSFL had a negative effect on the FI, FCR, and PI in the starter phase, consistent with previous studies reporting that the inclusion of BSFL in broiler chicken diets affects growth depending on the supplementation level. This effect may be attributed to chitin, an anti-nutritional factor present in insects [14]. During the initial phase (10–28 days), characterized by rapid growth, animals tend to consume more feed to compensate for the portion that is indigestible. By the grower and finisher phases, it was observed that the group fed with BSFL could improve production performance to match the control group, as indicated by factors such as the FCR and PI. This improvement may be attributed to older age and, accordingly, a more developed digestive system, enhancing digestion and nutrient absorption [23,24]. It has also been reported by Al Anas et al. [25] that chitinase levels in the gastrointestinal tract can be increased through diets containing chitin. Additionally, microorganisms can utilize chitin as a prebiotic [23,24]. A similar report suggested that using 10–20% BSFL can improve feed efficiency in broiler chickens [6,24,25,26].

The evaluation of economic returns is one of the most important indicators, as indigenous chickens have disadvantages such as a longer rearing period and lower feed efficiency, resulting in higher production costs than those of commercial chickens. However, the application of BSFL showed a decrease in FCG and did not affect performance. The application at a 12% level also resulted in the highest increase.

One of the outstanding features of TNC is its low fat content, which is in demand among consumers, especially those who are health-conscious [3]. In the previous study, we found a reduced response of the lipid compartment following the use of BSFL in the experimental diets. This response has a linear relationship, which may be due to chitin, which is a component of the external structure of BSFL [23]. Chitin is a prebiotic that increases the proportion of beneficial bacteria in the intestines and the production of butyrate, which is involved in fat metabolism [24]. In addition, the BSFL oil extraction process is conducted using the physical extraction method, which leaves behind the oil and lauric acid, the main constituent of the oil, which may influence the reduction in abdominal fat. Lauric acid, which is a medium-chain fatty acid (MCFA), enhances energy metabolism from fat and decreases the expression of genes associated with fat storage [26]. A previous study found that the application of BSFL oil increased taurine metabolites, which are involved in fat metabolism and total body fat storage, in TNC [4]. There are also studies reporting similar results; for example, Aprianto et al. [27] reported that supplementation with 1–3% BSF can reduce abdominal fat in broiler chickens. Nilugonda et al. [28] reported that 1–3% BSF reduced abdominal fat in layer hens, and Wang et al. [29] reported that supplementation with lauric acid derived from coconut demonstrated similar effects.

In this study, replacing soybean meal with BSFL at inclusion levels of 10% and 12% in animal diets resulted in increased concentrations of certain amino acids in breast meat, such as serine, glutamic acid, and threonine. This observation is consistent with the findings of Cullere et al. [16], who reported similar effects when partially substituting protein sources with 10% and 15% defatted BSFL in the diets of quail. Although the metabolic pathways leading to the accumulation of these amino acids in breast meat remain unclear, dietary supplementation with BSFL in this study also enhanced the levels of the aforementioned amino acids. These amino acids contribute significantly to the flavor and palatability of breast meat; however, these increases may still be below the threshold detectable [30]. Nevertheless, such findings may provide a basis for future studies aiming to improve the distinctive qualities of animal products. Furthermore, this study revealed that BSFL supplementation influenced the accumulation of arginine in breast meat. Although arginine is classified as a non-essential amino acid, it plays a crucial role in the synthesis of creatine [31], which is one of the key active compounds in poultry meat that supports prolonged and enhanced exercise performance in consumers [3,4]. Lysine, an essential amino acid important for muscle development and growth in humans [32], was also found to be increased. While total amino acid content was unaffected, dietary BSFL inclusion increased certain individual amino acids without negatively impacting others. These results suggest that using higher levels of BSFL in the diets of native Thai chickens is a promising strategy to enhance the nutritional and functional properties of poultry meat, warranting further investigation.

The substitution of soybean meal with BSFL in the diets (10% and 12%) altered the purine profile of TNC meat compared to the control diet. Chickens fed the BSFL diet showed slightly higher total purine content (mg/100 g) than those fed with the control diet, mainly due to higher adenine and guanine levels, while the change in hypoxanthine was relatively minor, resulting in slightly higher total purine levels than the control diet. This may be due to the higher digestibility of glutamic acid from BSFL than glutamic acid from soybean meal (24.3% and 3.6%, respectively) [33], which is involved in purine synthesis [34]. This is consistent with the report that higher levels of purines can accumulate in the tissues of chickens fed purine-rich diets (e.g., insects) [21]. From a human health perspective, such an increase in meat purines is noteworthy as dietary purines are metabolized into uric acid [35,36], and high purine intake is known to exacerbate hyperuricemia and gout in susceptible individuals [37]. Nevertheless, the absolute purine concentrations in all treatment groups remained within a moderate range (well below 200 mg/100 g) [38], comparable to conventional chicken meat and much lower than those found in organ meats or certain seafood. Thus, consuming chicken raised on BSFL diets would likely not markedly increase purine intake for the average consumer, and any slight purine elevation must be weighed against the nutritional benefits of BSFL as a sustainable, high-protein feed ingredient. Overall, this outcome aligns with prior observations that edible insects are generally purine-rich foods [37], indicating that while insect-based feeds may slightly raise purine content in meat, the resulting levels remain within safe limits for human consumption.

## 5. Conclusions

The inclusion of BSFL in Thai native chicken diets reduced production costs and improved economic returns. It significantly decreased abdominal fat without negatively affecting growth or carcass traits. BSFL enhanced the amino acid profile, increasing beneficial compounds like serine, glutamic acid, arginine, and lysine. Although purine content increased, levels remained within safe consumption limits. Overall, BSFL is a viable alternative to soybean meal for sustainable poultry production.

## Figures and Tables

**Table 1:** Composition and nutrient content of diets.

Ingredients (%)	Before the Experiment (1–9 Days)	Period 1 (10–28 Days)			Period 3 (43–70 Days)			Period 3 (43–70 Days)			
		Control	BSFL		Control	BSFL		Control	BSFL		
			10%	12%		10%	10%		T2	T3	
Corn meal	46.10	46.10	49.15	49.70	50.42	53.05	53.55	55.90	57.95	58.35	
Soybean meal	29.20	29.20	26.28	25.70	25.20	22.68	22.18	20.00	18.00	17.60	
BSFL ^1^	0.00	0.00	2.92	3.50	0.00	2.52	3.02	0.00	2.00	2.40	
Rice bran oil	2.10	2.10	0.75	0.50	3.28	2.05	1.80	3.80	2.90	2.70	
FFSB ^2^	18.00	18.00	16.30	16.00	16.50	15.10	14.85	16.30	15.15	14.95	
Choline chloride 60%	0.10	0.10	0.10	0.10	0.10	0.10	0.10	0.10	0.10	0.10	
Limestone	1.60	1.60	1.60	1.60	1.60	1.60	1.60	1.40	1.40	1.40	
Dicalciumphosphate%P21	1.80	1.80	1.80	1.80	1.80	1.80	1.80	1.40	1.40	1.40	
Premix ^3^	0.25	0.25	0.25	0.25	0.25	0.25	0.25	0.25	0.25	0.25	
Salt	0.40	0.40	0.40	0.40	0.40	0.40	0.40	0.40	0.40	0.40	
DL-Met	0.30	0.30	0.30	0.30	0.30	0.30	0.30	0.30	0.30	0.30	
Lysine	0.15	0.15	0.15	0.15	0.15	0.15	0.15	0.15	0.15	0.15	
Total	100.00	100.00	100.00	100.00	100.00	100.00	100.00	100.00	100.00	100.00	
**Chemical composition by analytic**											
CP, %	23.56	23.56	23.29	23.54	20.35	21.87	21.43	20.26	18.91	19.03	
GE, Kcal/kg	4.129	4.129	4.050	4.072	4.166	4.149	4.143	4.250	4.361	4.239	
Feed price (Baht/kg)	20.74	20.74	19.63	19.42	20.82	19.84	19.65	20.54	19.80	19.64	

^1^ BSFL: black soldier fly larvae; ^2^ FFSB: full-fat soybean; ^3^ provided per kilogram of diet, the formulation includes 4.80 million international units (MIU) of vitamin A, 2.00 MIU of vitamin D3, 30,000 international units (IU) of vitamin E, 1.20 g of vitamin K3, 1.20 g of vitamin B1, 3.20 g of vitamin B2, 2.00 g of vitamin B6, 0.0064 g of vitamin B12, 24.00 g of niacin, 0.80 g of folic acid, 0.08 g of biotin, and 6.00 g of pantothenic acid. Additionally, it contains 40.00 g of zinc (Zn), 48.00 g of manganese (Mn), 16.00 g of iron (Fe), 6.40 g of copper (Cu), 0.50 g of iodine (I), 0.04 g of cobalt (Co), and 0.12 g of selenium (Se), as well as 0.20 g of an antioxidant, 0.88 g of an anticaking agent, and 1.00 kg of carrier.

**Table 2:** Impact of dietary BSFL on the growth performance of TNC.

Performance	Control	BSFL		SEM	*p*-Value	Linear	Quadratic
		10%	12%				
Initial weight (g/b)	33.04	33.08	33.03	0.06	0.76	0.90	0.48
**Day 10–28**							
BW (g/b)	395.05	386.78	386.31	6.41	0.49	0.30	0.58
BWG (g/b)	361.96	353.69	353.22	6.41	0.49	0.30	0.58
FI ^1^ (g/b)	525.02 ^b^	671.64 ^a^	672.24 ^a^	9.65	<0.0001	<0.0001	<0.0001
FCR	1.45 ^b^	1.90 ^a^	1.90 ^a^	0.04	<0.0001	<0.0001	0.0002
SR ^2^ (%)	100.00	100.00	100.00	NA	NA	NA	NA
PI ^3^	138.68 ^a^	103.54 ^b^	103.28 ^b^	3.75	<0.0001	<0.0001	0.002
**Day 29–42**							
BW (g/b)	694.29	678.65	680.51	11.88	0.53	0.37	0.51
BWG (g/b)	299.24	291.87	294.20	15.38	0.92	0.80	0.77
FI ^1^ (g/b)	676.41	654.99	639.56	20.15	0.37	0.17	0.89
FCR	2.27	2.25	2.19	0.12	0.84	0.59	0.86
SR ^2^ (%)	100.00	100.00	100.00	NA	NA	NA	NA
PI ^3^	95.30	93.12	93.80	10.13	0.93	0.86	0.75
**Day 43–70**							
BW (g/b)	1361.68	1312.99	1350.81	19.37	0.16	0.68	0.08
BWG (g/b)	667.39	634.33	665.62	16.36	0.25	0.94	0.11
FI ^1^ (g/b)	1956.41 ^a^	1876.83 ^a^	1771.39 ^b^	29.93	0.009	0.003	0.71
FCR	2.87	2.97	2.66	0.12	0.20	0.23	0.14
SR ^2^ (%)	100.00	100.00	100.00	NA	NA	NA	NA
PI ^3^	86.64	79.87	92.72	4.65	0.42	0.38	0.11
**Day 10–70**							
BWG (g/b)	1328.59	1279.90	1317.72	19.37	0.16	0.68	0.08
FI ^1^ (g/b)	1908.60	1876.83	1771.39	43.89	0.11	0.09	0.46
FCR	1.44	1.47	1.35	0.04	0.10	0.10	0.10
SR ^2^ (%)	100.00	100.00	100.00	NA	NA	NA	NA
PI ^3^	154.34	145.74	163.46	5.04	0.08	0.21	0.17

The results are reported using the standard error of the mean (SEM) based on a sample size of four (n = 4). Means within rows that have different superscript letters (e.g., ^‘a, b’^) are significantly different at the *p* < 0.05 level. ^1^ FI = feed intake; ^2^ SR: survival rate; ^3^ PI: production index; NA = not applicable.

**Table 3:** Impact of dietary BSFL on economic aspects of TNC.

Economic Aspects	Control	BSFL		SEM	*p*-Value	Linear	Quadratic
		10%	12%				
Average feed price (USD/kg)	0.64	0.61	0.61	NA	NA	NA	NA
FCG (Baht/Bird)	1.22 ^a^	1.15 ^ab^	1.07 ^b^	0.03	0.01	0.003	0.96
SBR (Baht/Bird)	3.37	3.25	3.34	0.05	0.17	0.72	0.08
NPR (Baht/Bird)	2.15	2.10	2.27	0.05	0.08	0.09	0.08
ROI (%)	176.34 ^b^	183.79 ^b^	211.87 ^a^	6.88	0.01	0.005	0.21

The results are reported using the standard error of the mean (SEM) based on a sample size of four (n = 4). Means within rows that have different superscript letters (e.g., ^‘a, b’^) are significantly different at the *p* < 0.05 level; NA = not applicable.

**Table 4:** Impact of dietary BSFL on the carcass quality (%) of TNC.

Carcass	Control	BSFL		SEM	*p*-Value	Linear	Quadratic
		10%	12%				
**Dressing percentage (%)**	54.70	54.25	54.95	0.91	0.84	0.85	0.52
**Internal organs**							
Liver (%)	1.63	1.77	1.82	0.09	0.31	0.16	0.66
Heart (%)	0.46	0.44	0.43	0.02	0.48	0.24	0.97
Pancreas (%)	0.18	0.18	0.16	0.01	0.56	0.44	0.43
Spleen (%)	0.18	0.21	0.22	0.03	0.57	0.36	0.68
Gizzards (%)	2.19	2.40	2.28	0.15	0.55	0.68	0.35
Abdominal fat (%)	0.32 ^a^	0.19 ^ab^	0.12 ^b^	0.06	0.05	0.02	0.67
**External organs**							
Wing (%)	9.96	10.15	10.24	0.15	0.34	0.16	0.12
Thigh (%)	13.43	13.13	13.58	0.41	0.69	0.79	0.42
Inner fillet (%)	3.72	3.53	3.71	0.12	0.38	0.99	0.18
Outer fillet (%)	11.48	11.12	11.17	0.45	0.80	0.62	0.70
Drumstick (%)	11.18	11.02	11.17	0.17	0.72	0.99	0.43
**Edible meat (%)**	49.75	49.73	49.93	0.75	0.98	0.86	0.91

The results are reported using the standard error of the mean (SEM) based on a sample size of four (n = 4). Means within rows that have different superscript letters (e.g., ^‘a, b’^) are significantly different at the *p* < 0.05 level.

**Table 5:** Impact of dietary BSFL on the meat quality of TNC.

Quality of Breast Meat	Control	BSFL		SEM	*p*-Value	Linear	Quadratic
		10%	12%				
**Color**							
Lightness (L*)	44.08	41.37	44.50	2.25	0.36	0.62	0.19
Redness (a*)	1.46	1.44	0.96	0.45	0.76	0.51	0.71
Yellowness (b*)	7.15	8.51	8.08	0.53	0.24	0.19	0.26
**Texture profile analysis**							
Hardness (g)	730.3	845.8	785.3	124.93	0.76	0.73	0.52
Springiness (g)	0.59	0.60	0.61	0.03	0.92	0.69	0.94
Cohesiveness (g)	0.65	0.63	0.64	0.02	0.55	0.79	0.30
Gumminess (g)	475.10	535.50	506.10	87.25	0.85	0.78	0.64
Chewiness (g)	292.51	325.18	308.03	55.28	0.89	0.82	0.68

The results are reported using the standard error of the mean (SEM) based on a sample size of four (n = 4).

**Table 6:** Impact of dietary BSFL on the amino acid profile of breast meat of TNC.

Amino Acid (g/100 g of Breast Meat)	Control	BSFL		SEM	*p*-Value	Linear	Quadratic
		10%	12%				
**Non-essential amino acids**							
Serine	0.57 ^c^	0.79 ^a^	0.67 ^b^	0.051	0.025	0.139	0.0142
Glutamic	3.41 ^b^	3.81 ^a^	3.61 ^ab^	0.008	<0.0001	<0.0001	<0.0001
Alanine	0.68	0.68	0.71	0.039	0.775	0.576	0.684
Cysteine	1.21	1.20	1.24	0.019	0.133	0.227	0.094
Tyrosine	0.81	0.78	0.83	0.028	0.542	0.491	0.400
Arginine	3.58 ^b^	3.72 ^a^	3.75 ^a^	0.040	0.021	0.009	0.284
**Total Non-essential amino acids**	10.26 ^b^	10.98 ^a^	10.81^a^	0.170	0.023	0.032	0.036
**Essential amino acids**							
Threonine	2.77 ^c^	3.03 ^a^	2.91 ^b^	0.030	0.0008	0.0075	0.0006
Valine	0.66	0.73	0.74	0.035	0.199	0.101	0.494
Isoleucine	1.88	1.83	1.94	0.037	0.085	0.136	0.073
Leucine	3.75	3.76	3.81	0.025	0.189	0.081	0.797
Phenylalanine	2.24	2.19	2.26	0.042	0.449	0.743	0.238
Lysine	3.92 ^b^	4.00 ^a^	4.01 ^a^	0.008	<0.0001	<0.0001	0.005
Histidine	1.78	1.91	1.79	0.055	0.310	0.801	0.145
**Total essential amino acids**	17.00	17.45	17.46	0.218	0.181	0.106	0.360
**Total amino acid**	27.25	28.42	28.29	0.388	0.074	0.061	0.133

The results are reported using the standard error of the mean (SEM) based on a sample size of three (n = 3). Means within rows that have different superscript letters (e.g., ^‘a–c’^) are significantly different at the *p* < 0.05 level.

**Table 7:** Impact of dietary BSFL on purine content of breast meat of TNC.

Purine Content (g/100 g of Breast Meat)	Control	BSFL		SEM	*p*-Value	Linear	Quadratic
		10%	12%				
Purine	35.12 ^c^	107.40 ^a^	89.58 ^b^	0.315	<0.0001	<0.0001	<0.0001
Adenine	10.40 ^b^	12.09 ^a^	12.16 ^a^	0.184	0.0003	0.0002	0.0051
Guanine	6.28 ^b^	9.93 ^a^	8.98 ^a^	0.489	0.0016	0.0030	0.0033
Hypoxanthine	18.44 ^c^	85.12 ^a^	68.44 ^b^	0.281	<0.0001	<0.0001	<0.0001

The results are reported using the standard error of the mean (SEM) based on a sample size of three (n = 3). Means within rows that have different superscript letters (e.g., ^‘a–c’^) are significantly different at the *p* < 0.05 level.

## Data Availability

The supporting data for this study are included within the article. For further information or inquiries about the original contributions, please contact the corresponding author.
